# Mutation of the Surface Layer Protein SlpB Has Pleiotropic Effects in the Probiotic *Propionibacterium freudenreichii* CIRM-BIA 129

**DOI:** 10.3389/fmicb.2018.01807

**Published:** 2018-08-17

**Authors:** Fillipe L. R. do Carmo, Wanderson M. Silva, Guilherme C. Tavares, Izabela C. Ibraim, Barbara F. Cordeiro, Emiliano R. Oliveira, Houem Rabah, Chantal Cauty, Sara H. da Silva, Marcus V. Canário Viana, Ana C. B. Caetano, Roselane G. dos Santos, Rodrigo D. de Oliveira Carvalho, Julien Jardin, Felipe L. Pereira, Edson L. Folador, Yves Le Loir, Henrique C. P. Figueiredo, Gwénaël Jan, Vasco Azevedo

**Affiliations:** ^1^Departamento de Biologia Geral, Instituto de Ciências Biológicas, Universidade Federal de Minas Gerais, Belo Horizonte, Brazil; ^2^Institut National de la Recherche Agronomique, UMR1253 Science & Technologie du Lait & de l'Oeuf, Rennes, France; ^3^Agrocampus Ouest, UMR1253 Science & Technologie du Lait & de l'Oeuf, Rennes, France; ^4^Instituto de Biotecnología, CICVyA - Instituto Nacional de Tecnología Agropecuaria, Hurlingham, Argentina; ^5^Consejo Nacional de Investigaciones Científicas y Técnicas, Buenos Aires, Argentina; ^6^AQUACEN, Escola de Veterinária, Universidade Federal de Minas Gerais, Belo Horizonte, Brazil; ^7^Departamento de Biointeração do Instituto de Ciências da Saúde, Universidade Federal da Bahia, Salvador, Brazil; ^8^Centro de Biotecnologia, Universidade Federal da Paraíba, João Pessoa, Brazil

**Keywords:** bacteria genomic, bacteria proteomic, surface layer protein, HDMSE, shotgun proteomic

## Abstract

*Propionibacterium freudenreichii* is a beneficial Gram-positive bacterium, traditionally used as a cheese-ripening starter, and currently considered as an emerging probiotic. As an example, the *P. freudenreichii* CIRM-BIA 129 strain recently revealed promising immunomodulatory properties. Its consumption accordingly exerts healing effects in different animal models of colitis, suggesting a potent role in the context of inflammatory bowel diseases. This anti-inflammatory effect depends on surface layer proteins (SLPs). SLPs may be involved in key functions in probiotics, such as persistence within the gut, adhesion to host cells and mucus, or immunomodulation. Several SLPs coexist in *P. freudenreichii* CIRM-BIA 129 and mediate immunomodulation and adhesion. A mutant *P. freudenreichii* CIRM-BIA 129Δ*slpB* (CB129Δ*slpB*) strain was shown to exhibit decreased adhesion to intestinal epithelial cells. In the present study, we thoroughly analyzed the impact of this mutation on cellular properties. Firstly, we investigated alterations of surface properties in CB129Δ*slpB*. Surface extractable proteins, surface charges (ζ-potential) and surface hydrophobicity were affected by the mutation. Whole-cell proteomics, using high definition mass spectrometry, identified 1,288 quantifiable proteins in the wild-type strain, i.e., 53% of the theoretical proteome predicted according to *P. freudenreichii* CIRM-BIA 129 genome sequence. In the mutant strain, we detected 1,252 proteins, including 1,227 proteins in common with the wild-type strain. Comparative quantitative analysis revealed 97 proteins with significant differences between wild-type and mutant strains. These proteins are involved in various cellular process like signaling, metabolism, and DNA repair and replication. Finally, *in silico* analysis predicted that *slpB* gene is not part of an operon, thus not affecting the downstream genes after gene knockout. This study, in accordance with the various roles attributed in the literature to SLPs, revealed a pleiotropic effect of a single *slpB* mutation, in the probiotic *P. freudenreichii*. This suggests that SlpB may be at a central node of cellular processes and confirms that both nature and amount of SLPs, which are highly variable within the *P. freudenreichii* species, determine the probiotic abilities of strains.

## Introduction

Probiotic bacteria are defined as “living microorganisms which when administered in adequate amounts confer a health benefit on the host” (Food and Agriculture Organization of the United Nations and World Health Organization, 2002). This term was further used by International Scientific Association for Probiotics and Prebiotics (ISAP) (Hill et al., 2014). Clinical proofs of efficiency were indeed obtained, in the context of antibiotic- and *Clostridium difficile*-associated diarrhea (Rondanelli et al., 2017), lactose intolerance (Oak and Jha, 2018), irritable bowel syndrome (IBS) (Ford et al., 2014), and ulcerative colitis, one of the disorders that constitute Inflammatory bowel disease (IBD) (Plaza-Díaz et al., 2017). The mechanisms underpinning these effects mainly belong to three categories: (i) metabolic effects, (ii) modulation of the gut microbiota, and (iii) probiotic/host molecular interactions. Although lactobacilli and bifidobacteria were mainly considered for probiotic usage, promising effects were also reported for dairy propionibacteria (Rabah et al., 2017).

The probiotic properties of dairy propionibacteria are strain-dependent and include microbiota modulation, apoptosis modulation in colonic cells and immunomodulation. Some of these probiotic abilities were validated at the clinical level. Microbiota modulation by dairy propionibacteria result in a bifidogenic effect (Roland et al., 1998; Seki et al., 2004; Suzuki et al., 2006). The corresponding molecular mechanisms were elucidated, and two molecules are shown to be involved in bifidogenic effect: 1,4-dihydroxy-2-naphtoic acid (DHNA) and 2-amino-3-carboxy-1,4-naphthoquinone (ACNQ) (Isawa et al., 2002; Furuichi et al., 2006). The pro-apoptotic effect of dairy propionibacteria was evidenced using *in vitro* cellular models (Jan et al., 2002) and animals models (Lan et al., 2008). This effect is mainly due to the production of the short chain fatty acids (SCFA) acetate and propionate by dairy propionibacteria (Lan et al., 2007; Cousin et al., 2016). The anti-inflammatory effect was suggested in IBD patients (Mitsuyama et al., 2007) and confirmed in animal colitis models (Foligné et al., 2010; Plé et al., 2015, 2016). Immunomodulatory properties are due to several metabolites as SCFAs and to cells wall component (Rabah et al., 2017). Indeed, surface proteins considered as microorganism-associated molecular patterns (MAMP) play a pivotal role in interaction with host's immune system (Deutsch et al., 2012; Le Maréchal et al., 2015). This includes SlpB and SlpE, surface proteins anchored to the cell wall via surface-layer homology (SLH) domains (Deutsch et al., 2017; do Carmo et al., 2018). Indeed, mutation of *slpB* and *slpE* genes clearly affected the immunomodulatory properties of *P. freudenreichii* (Deutsch et al., 2017). We have recently shown that SlpB is involved both in immunomodulation and in adhesion to cultured human intestinal epithelial cells (do Carmo et al., 2017).

In probiotic bacteria, extractable surface proteins play several role in bacterium/host interaction, protection against environmental stresses, inhibition of pathogens, survival within the host digestive tract, and determination or maintenance of cell shape (Hynönen and Palva, 2013; do Carmo et al., 2018). In this study, we investigated the impact of *slpB* gene mutation on the physiology of *P. freudenreichii* CIRM-BIA 129 using a proteomic approach. In this purpose, we investigated alterations in extractable surface proteins and in the whole-cell proteome. We compared wild-type CIRM-BIA 129 with mutant CB129Δ*slpB*. We report pleiotropic effects of this single mutation on physicochemical properties of this propionibacteria.

## Materials and methods

### Bacterial strains and culture conditions

The wild-type *P. freudenreichii* CIRM-BIA 129 (WT) strain and genetically modified *P. freudenreichii* CIRM-BIA 129Δ*slpB* strain (CB129Δ*slpB*) (do Carmo et al., 2017) were grown at 30°C in Yeast Extract Lactate (YEL) broth (Malik et al., 1968). For the CB129Δ*slpB*, YEL culture media were supplemented with chloramphenicol (10 μg.mL^−1^). The growth of *P. freudenreichii* strains was monitored spectrophotometrically by measuring the optical density at 650 nm (OD_650 nm_), as well as by counting colony-forming units (CFUs) in YEL medium containing 1.5% agar. *P. freudenreichii* strains were harvested in a stationary phase (76 h, 2 × 10^9^ CFU.mL^−1^, determined by plate counts) by centrifugation (8,000 × g, 10 min, 4°C).

### Inventory of extractable surface proteins using guanidine hydrochloride and MS/MS

Proteins were guanidine-extracted, trypsinolysed and subjected to mass spectrometry (Le Maréchal et al., 2015). Peptides were separated by Nano-LC-MS/MS using a Dionex U3000-RSLC nano-LC system fitted to a Qexactive mass spetrometer (Thermo Scientific, San Jose, CA, USA) equipped with a nano-electrospray ion source (ESI) (Proxeon Biosystems A/S, Odense, Denmark). Peptides were identified from MS/MS spectra using the X!Tandem pipeline 3.4.3 software (Langella et al., 2017) for search into two concatenated databases: (i) a homemade database containing all the predicted proteins of the *P. freudenreichii* CIRM-BIA 129 used in this study and (ii) a portion of the UniProtKB database corresponding to taxonomy 754252: *P. freudenreichii* subsp. *shermanii* (strain ATCC 9614/CIP 103027/CIRM-BIA1).

### Zeta potential analysis

Electrophoretic mobility (zeta potential) was determined according to the well-described protocol of Schär-Zammaretti and Ubbink (2003). Bacteria were harvested from a 5 mL stationary phase culture by centrifugation (8.000 × g, 10 min, room temperature) and washed twice with a PBS buffer pH 7.0. Cell count of the final suspensions was approximately 10^8^ CFU/ml. The pellet was resuspended in a 10 mM KH_2_PO_4_ solution (pH 7.0). The electrophoretic mobility was measured by using a ZetaSizer nanoZS (Malvern Instruments, Malvern, United Kingdom) and a glass capillary Zetasizer Nanoseries DTS 1061 (Malvern Instruments, Malvern, United Kingdom) as the electrophoretic cell. Electrophoretic mobilities were converted to the ζ-potential using the Helmholtz-Smoluchowski equation (Schär-Zammaretti and Ubbink, 2003). All experiments were done in biological and technical triplicates.

### Cell surface hydrophobicity analysis

The Microbial Adhesion To Hydrocarbons (MATH) assay was performed as described by Kos et al. (2003). The optical density of the stationary phase bacteria was adjusted to an OD_650 nm_ = 1. The samples were centrifuged for 5 min, 10,000 × g at room temperature and the pellets washed twice with the same volume of PBS pH 7.0 prior to resuspension in 15 mL of 0.1M KNO_3_, pH 6.2. An aliquot of each bacterial suspension (4 ml) was mixed with 1 mL of the solvent (Xylene, chloroform and ethyl acetate), incubated for 5 min at room temperature and mixed by vortex during 120 s. Subsequently, samples were incubated during 60 min to allow phases separation, the aqueous phase was carefully removed and absorbance (OD_600 nm)_ was determined as above. Cell surface hydrophobicity in terms of per cent (H %) was calculated using the following formula: H % = (1–A1/A0) × 100. All experiments were done in biological and technical triplicates.

### Transmission electron microscopy assay

Cultures were grown on YEL medium to an OD_650 nm_ of 1. Transmission electron microscopy was executed after bacteria were washed with PBS and fixed overnight at 4°C in 0.1 M sodium cacodylate buffer (pH 7.2) containing 2% glutaraldehyde. Fixed bacteria were rinsed and stored at 4°C in cacodylate buffer containing 0.2 M sucrose. They were then postfixed with 1% osmium tetroxide containing 1.5% potassium cyanoferrate and 2% uranyl acetate in water before gradual dehydration in ethanol (30% to 100%) and embedding in Epon. Thin sections (70 nm) were collected on 200-mesh cooper grids and counterstained with lead citrate before examination. The thickness of the cell wall was determined using the imageJ software in both strains analyzed by Transmission Electron Microscopy (TEM) as described (Foligné et al., 2010; Deutsch et al., 2012).

### Stress conditions challenge

*P. freudenreichii* strains in stationary phase were subjected to lethal doses of different stresses. The acid challenge was carried out at pH 2.0 for 1 h as described (Jan et al., 2000). The bile salts stress was induced by adding 1.0 g/l of bile salts for 60 s as described (Leverrier et al., 2003). For the thermic stress, bacteria were heated for 30 min at 63°C. Viable cells were determined by serial dilutions of samples made up in peptone water (0.1% bacteriological peptone, Kasvi, Brazil), adjusted to pH 7.0 and containing 0.9% NaCl, into YEL medium containing 1.5% agar. CFU were counted after 6 days of anoxic incubation at 30°C (Anaerocult® A - Merck Millipore). All experiments were done in biological and technical triplicates.

### Whole-cell protein extraction and preparation of total bacterial lysates

The optical density of the stationary phase bacteria was adjusted to an OD_650 nm_ = 1. The cultures were centrifuged for 5 min, 10,000 × g at room temperature and the bacterial pellets from biological triplicates were resuspended in 1 mL of lysis buffer containing 42% urea, 15% thiourea, 4% SDC (sodium deoxycholate), 12.5 mM Tris-HCl pH 7.5 and 1.5% dithiothreitol (DTT) with 10 μL of protease inhibitor (GE HealthCare, Pittsburgh, USA). Next, whole-cell proteins were extracted as described (Silva et al., 2014) and quantified by Qubit 2.0 fluorometer (Invitrogen, Carlsbad, USA). 100 μg of each protein extract were denatured with 0.2% of RapiGest SF solution (Waters, Milford, USA) at 80°C for 15 min, reduced with 100 mM DTT at 60°C for 30 min, and alkylated with 300 mM iodoacetamide at room temperature in a dark room for 30 min (Leibowitz et al., 2017). Subsequently, proteins were enzymatically digested with 10 μl of trypsin at 0.5 μg.μL^−1^ (Promega, Madison, USA), and the digestion stopped with the addition of 10 μL of 5% trifluoroacetic acid (TFA) (Sigma Aldrich, Saint Louis, USA) (Silva et al., 2017). Tryptic peptides were subjected to SDC removal (Lin et al., 2010), desalted using C18 MacroSpin Columns (Harvard Apparatus, Holliston, USA), according to the manufacturer's instructions, and dried under vacuum in the Eppendorf™ Vacufuge™ Concentrator (Eppendorf, Hamburg, Germany) (Wong et al., 2013). Prior to injection, the peptides were resuspended in 20 mM ammonium formate (Sigma Aldrich) and transferred to Waters Total Recovery vials (Waters).

### LC-HDMS^E^ analysis and data processing

Quantitative proteomics analyses were conducted with Bidimensional Nano Ultra-Performance Liquid Chromatography (nanoUPLC) tandem Nano Electrospray High Definition Mass Spectrometry (nanoESI-HDMS^E^) both using a 1-h reverse-phase (RP) gradient from 7 to 40% (v/v) acetonitrile (0.1% v/v formic acid) and a 500 nL.min^−1^ nanoACQUITY UPLC 2D Technology system (Waters) (Gilar et al., 2005). A nanoACQUITY UPLC High Strength Silica (HSS) T3 1.8 μm, 75 μm × 150 mm column (pH 3) was used in conjunction with a RP Acquity UPLC Nano Ease XBridge BEH130 C18 5 μm, 300 μm × 50 mm nanoflow column (pH 10) (Silva et al., 2017). Typical on-column sample loads were 500 ng of total protein digests for each sample of the 5 fractions (500 ng per fraction/load).

The measurements for all samples by mass spectrometer was operated in resolution mode with a typical m/z resolving power of at least 25,000 Full Width at Half Maximum (FWHM) and an ion mobility cell that was filled with helium gas and a cross-section resolving power at least 40 Ω/Δ Ω. The effective resolution with the conjoined ion mobility was 25,000 FWHM. Analyses were performed using nano-electrospray ionization in positive ion mode nanoESI (+) and a NanoLock-Spray (Waters) ionization source. The lock mass channel was sampled every 30 s. The mass spectrometer was calibrated with an MS/MS spectrum of [Glu1]-Fibrinopeptide B human (Glu-Fib) solution (100 fmol.μL^−1^) that was delivered through the reference sprayer of the NanoLock-Spray source. The double-charged ion ([M + 2H]^2+^ = 785.8426) was used for initial single-point calibration, and MS/MS fragment ions of Glu-Fib were used to obtain the final instrument calibration.

The multiplexed data-independent acquisition (DIA) scanning with added specificity and selectivity of a non-linear “T-wave” ion mobility (HDMS^E^) device was performed with a Synapt G2-Si HDMS mass spectrometer (Waters) (Giles et al., 2011). Synapt G2-Si HDMS was automatically planned to switch between standard MS (3 eV) and elevated collision energies HDMS^E^ (19–45 eV) applied to the transfer “T-wave” collision-induced dissociation cell with nitrogen gas. The trap collision cell was adjusted to 1 eV, using a millisecond scan time that was previously adjusted based on the linear velocity of the chromatographic peak that was delivered through nanoACQUITY UPLC (Waters). A minimum of 20 scan points was generated for each single peak, both in low-energy and high-energy transmission at an orthogonal acceleration time-of-flight (oa-TOF) and a mass range from m/z 50 to 2,000.

Mass spectrometric analysis of tryptic peptides was performed using a mass spectrometer equipped with a T-Wave-IMS device (Waters) in MS^E^ mode following the method previously described (Distler et al., 2014). Stoichiometric measurements based on scouting runs of the integrated total ion account prior to analysis were performed to ensure standardized molar values across all samples. Therefore, the tryptic peptides of each strain were injected with the same amount on the column. The radio frequency (RF) offset (MS profile) was adjusted such that the nanoESI-HDMS^E^ data were effectively acquired from m/z 400 to 2000, which ensured that any masses less than m/z 400 that were observed in the high energy spectra with arose from dissociations in the collision cell (Silva et al., 2017).

The mass spectrometry proteomics data have been deposited to the ProteomeXchange Consortium via the PRIDE (Vizcaíno et al., 2016) partner repository with the dataset identifier PXD009804.

### Proteins identification and quantification

HDMS^E^ raw data were processed using Progenesis QI for Proteomics (QIP) v.2.0 (Nonlinear Dynamics, Newcastle, UK) as described by Kuharev et al. (2015). For proteins identification, the peptides were searching against a *P. freudenreichii* strain CIRM-BIA 129 database as described above. The reversed sequences were joined together to the original sequences using ProteinLynx Global Server (PLGS) v 3.0.2 (Waters) database management tool. The reversed sequences were used to calculate the false positive rate during identification process. Next, the following parameters were used for peptide identification: digest reagent = trypsin; maximum missed cleavage = one; maximum protein mass = 600 kDa; modifications: carbamidomethyl of cysteine (fixed), acetyl N-terminal (variable), phosphoryl (variable), oxidation of methionine (variable); search tolerance parameters: peptide tolerance = 10 ppm, fragment tolerance = 20 ppm, maximum false discovery rate (FDR) = 1%.

The protein-level quantitation was performed with Relative Quantitation using Hi-N algorithm. Proteins identified with at least two peptides and presents in at least two of the three biological replicates were considered (Silva et al., 2014). The proteins list was exported by the function “export protein measurements” and was used to subsequent bioinformatics analysis. Proteins were considered to be differentially expressed between mutant and wild type if there were a significant (*p* < 0,05, ANOVA) change in expression ≥ 2-fold (log2 ratio ≥ 1.0). A volcano plot was generated to visualize the differentially expressed proteins across these strains.

### Extraction of genomic DNA of the CB129Δ*slpB* strain

Genomic DNA was extracted from CB129Δ*slpB* culture grown in YEL medium supplemented with chloramphenicol (10 μg ml^−1^), during the phase (76 h at 30°C). Samples was centrifuged at 4°C and 8,000 × g for 10 min. Bacterial pellets were resuspended in 1 ml Tris/EDTA/RNase [10 mM Tris/HCl (pH 7.0), 10 mM EDTA (pH 8.0), 300 mM NaCl, 50 μg RNase A ml^−1^] with 50 mg of Glass beads VK01 and cell lysis occurred in Precellys®24 by 2 cylces of 15 s at 6,500 rpm. DNA was purified using phenol/chloroform/isoamyl alcohol and precipitated with ethanol according with Sambrook and Russell (2001). DNA concentrations were determined spectrophotometrically in Thermo Scientific NanoDrop 1000.

### Genome sequencing, assembly and annotation of the CB129Δ*slpB* strain

CB129Δ*slpB* strain sequencing libraries were constructed using 100 ng of genomic DNA. The gDNA was sheared with the Ion Shear™ Plus Reagents Kit and barcoded using the Ion Xpress Fragment Library kit and Ion Xpress™ Barcode Adapters (Life Technologies, USA), according to the manufacturer's protocol. Size selection of ~400 bp was performed with 2% E-Gel® SizeSelect™ Agarose Gels (Invitrogen, USA) and quantified with the Ion Library Quantitation Kit. The libraries were amplified with the OneTouch Template 400 kit on the Ion One Touch™ 2 (Life Technologies) and enriched on the Ion OneTouch™ ES (Life Technologies). Genomic libraries were enriched using Ion PI™ Hi-Q™ Sequencing Polymerase in the Ion 318™ v2 Chip, according to the manufacturer's protocols, and they were sequenced using Ion Torrent Personal Genome Machine (PGM). The amplification processes were performed using Ion PGM™ Hi-Q™ Sequencing 400 Polymerase with required 1,100 flows. Finally, signal processing was performed using Torrent Suite 4.2.1 to conclude the sequencing process.

*De novo* assembly was conducted using the software Newbler v 2.9 (Roche 454, USA). The assembled contigs were oriented to generate a scaffold using CONTIGuator v 2.7 (Galardini et al., 2011) and the strains *P. freudenreichii* CIRM-BIA 1 (FN806773.1) and *P. freudenreichii* JS17 (LT618789) as reference. The *P. freudenreichii* CIRM-BIA 1 strain (without the *slpB* gene) was used for comparative analysis as it is a reference from INRA strain collection strain and *P. freudenreichii* JS17 strain was used due to the presence of the s-layer gene *slpB*. CLC Genomics Workbench 7.0 (Qiagen, USA) was used to map the raw reads against the reference genome and to generate the consensus sequence used to the gap filling. The plasmid that integrated within and disrupted the *slpB* gene was not found in the scaffold, but its sequence was found within the contigs that were excluded during the scaffold generation. It was manually inserted to the scaffold by mapping its ends on the *slpB* gene and using the overlap sequences as coordinates for the insertion. The insertion was validated by mapping the reads on the assembly and checking for mismatches on the regions flanking the plasmid. The genome of CB129Δ*slpB* strain was annotated automatically using RAST pipeline (Aziz et al., 2008; Brettin et al., 2015).

### Bioinformatics analyses

The predicted proteins of CB129Δ*slpB* and WT strain were analyzed using the SurfG+ v1.0 tool (Barinov et al., 2009) to predict sub-cellular localization. It enabled the classification of proteins within the following categories: cytoplasmic (CYT), membrane (MEM), potentially surface-exposed (PSE) and secreted (SEC). The prediction of orthologous groups by functional category the sequences was performed using Cluster of Orthologous Genes (COG) database version 2014db (Galperin et al., 2015). The COG database search was performed using an *in-house* script (available at https://github.com/aquacen/blast_cog). The number of predicted proteins in relation to subcellular localization and functional category were visualized in plots generated using TIBCO SpotFire software 7.0 (TIBCO, Boston, USA) from the protein list exported of QIP. The Interactivenn web-based tool (Heberle et al., 2015) was used to evaluate the shared proteins among strains through Venn diagram.

Protein-protein interaction (PPI) network was constructed using interolog mapping methodology and metrics according to Folador et al. (2014). To generate a preview of the interaction network was generated using Cytoscape version 2.8.3 (Shannon et al., 2003) with a spring-embedded layout. To indicate the reliability of our predicted PPIs in the database STRING, the network was selected using the score 500 (0.5). In the PPI network, the interactions with score close to 500 are with red or yellow lines and, above 700 in dark green lines. The score indicating how much the pair of proteins in the interaction is similar (homologous) to the interaction according to the database. In the PPI, they interact with at least 65% identity with at least 65% coverage.

A circular map comparing the chromosome of CB129Δ*slpB* with *P. freudenreichii* CIRM-BIA 1 and JS17 strains was generated using BLAST Ring Image Generator(BRIG) software v0.95 (Alikhan et al., 2011). Operon prediction in CB129Δ*slpB* strain was performed using FGENESB (http://www.softberry.com).

### Statistical analyses

Growth curve, MATH assay, Zeta potential measure, and stress challenges were performed with three technical replicates and three biological replicates. The results were expressed as means ± standard deviations. Statistical analyses were performed in GraphPad Prism Software version 7 (GraphPad Software) using Student's *t*-test, one-way or two-way ANOVA with SIDAK's or Tukey *post-hoc* analyses for multiple comparisons. Asterisks represent statistically significant differences and were indicated as follows: ^*^p < 0.05; ^**^p < 0.01; ^***^p < 0.001.

## Results

### Impact of *slpB* mutation on *P. freudenreichii* extractable surface proteins

SLPs play a key role in probiotic/host interactions and we have shown that such interactions are impaired in an *slpB* mutant. Electrophoretic analysis of guanidine extracts confirmed the disappearance of the corresponding SlpB protein (do Carmo et al., 2017). In the present study, we further investigated these extractable fractions in order to decipher the impact of such a single mutation on the inventory of SLPs, and more widely, of extractable surface proteins, including surface layer associated proteins (SLAPs). Using nanoLC-MS/MS, we identified 40 surface extractable proteins in CB129Δ*slpB* strain, yet 33 in the parental wild-type CIRM BIA 129 one (Table 1). The core of extractable proteins, non-covalently bound to the cell wall, common to mutant and parental strains, was composed of 23 proteins, including solute-binding protein of the ABC transport system (BopA), internalin A (InlA), surface protein with SLH Domain E (SlpE), and surface-Layer Protein A (SlpA). Moreover, it comprised a series of cytoplasmic proteins involved in different biological processes like Heat shock 70 kDa protein 1 (HSP70 1), Clp chaperone, GroL1 and GroL2, Elongation factor Tu, and subunits of Methylmalonyl-CoA mutase and subunits of Methylmalonyl-CoA carboxytransferase. Among extractable proteins specific of the CB129Δ*slpB*, we identified proteins involved in metabolic processes like Coenzyme A transferase involved in acetyl-CoA metabolic process and Pyruvate phosphate dikinase Pyruvate synthase involved in pyruvate metabolic process. Furthermore, this specific subset also comprised another protein involved in stress response (HSP70 2). As expected, the SlpB protein was found only in the parental wild type CIRM BIA 129, yet not in the CB129Δ*slpB* mutant.

**Table 1:** Proteins identified in the extraction of surface proteins non-covalently bound to the cell wall using guanidine hydrochloride of CB 129 wild-type and CB129Δ*slpB* strains^1^.

**Strain**						**Wild-type**						**CB129**Δ***slpB***				
**Group ID^a^**	**Sub-group ID^b^**	**locus_tag**	**Protein description^c^**	**SurfG+^d^**	**COG letter^e^**	**MW^f^**	**log(*e*-value)^g^**	**Coverage^h^**	**Uniques^i^**	**Specific uniques^j^**	**emPAI^k^**	**log(*e*-value)^g^**	**Coverage^h^**	**Uniques^i^**	**Specific uniques^j^**	**emPAI^k^**
a1	a1.a1	PFCIRM129_05460	Surface protein with SLH domain (S-layer protein E)	SEC	O	59.2	−125.6	50	19	18	47.3	−138.8	55	18	17	41.8
a1	a1.a2	PFCIRM129_00700	Surface layer protein B (S-layer protein B)	SEC	O	56.8	−263.2	74	34	33	37274.9	–	–	–	–	–
a1	a1.a3	PFCIRM129_09350	Surface layer protein A (S-layer protein A)	SEC	O	58.3	−174.3	75	24	23	16.0	−143.5	68	22	21	9.0
a2	a2.a1	PFCIRM129_12235	Internaline A	SEC	S	145.5	−464.8	67	53	–	89.1	−426.3	65	49	–	42.3
a3	a3.a1	PFCIRM129_03680 & PFCIRM129_03685	MERGED = TRUE	–	–	95.9	−186.7	43	18	–	283.8	−196.6	47	20	–	431.9
a4	a4.a1	PFCIRM129_10100	60 kDa chaperonin 2 (Protein Cpn60 2) (groEL protein 2) (Heat shock protein 60 2)	CYT	O	56.4	−82.5	38	13	–	5.3	−112.6	49	18	–	14.8
a5	a5.a1	PFCIRM129_07835	60 kDa chaperonin 1 (Protein Cpn60 1) (groEL protein 1) (Heat shock protein 60 1)	CYT	O	56	−89.2	38	12	–	2.2	−134.7	60	21	–	7.5
a6	a6.a1	PFCIRM129_06355	Chaperone clpB 2 (ATP-dependent Clp protease B2) (Clp chaperone)	CYT	O	94.2	−39.6	19	10	9	1.2	−103.0	29	18	–	3.0
a7	a7.a1	PFCIRM129_06315	Chaperone protein dnaK 1 (Heat shock protein 70 1) (Heat shock 70 kDa protein 1) (HSP70 1)	CYT	O	65.3	−24.0	13	5		0.6	−61.2	34	15	12	3.8
a7	a7.a2	PFCIRM129_08775	Chaperone protein dnaK 2 (Heat shock protein 70 2) (Heat shock 70 kDa protein 2) (HSP70 2)	CYT	O	67.1	–	–	–	–	–	−43.7	23	10	7	2.0
a9	a9.a1	PFCIRM129_08275	Elongation factor Tu	CYT	J	43.6	−43.7	33	7	–	2.0	−32.4	28	7	–	3.4
b11	b11.a1	PFCIRM129_11405	30S ribosomal protein S1	CYT	J	53.5	−5.4	7	2	–	0.3	−58.9	27	8	–	1.6
b12	b12.a1	PFREUD_01840	Pyruvate synthase/Pyruvate-flavodoxin oxidoreductase	CYT	C	136.4	–	–	–	–	–	−67.5	16	14	–	1.2
b13	b13.a1	PFCIRM129_10305	Methylmalonyl-CoA carboxytransferase 5S subunit. (transcarboxylase 5S) 505 bp	CYT	C	55.5	−23.3	16	5	–	0.7	−37.1	23	9	–	1.8
b14	b14.a1	PFCIRM129_06950	Trigger factor (TF)	CYT	O	57.3	−8.3	6	2	–	0.3	−36.7	20	6	–	2.0
b15	b15.a1	PFCIRM129_07240	Methylmalonyl-CoA mutase large subunit (Methylmalonyl-CoA mutase alpha subunit) (MCM-alpha) (MUTB-(R)-2-Methyl-3-oxopropanoyl-CoA CoA-carbonylmutase)	CYT	I	80.1	−15.6	7	4	–	0.4	−38.3	15	8	–	1.0
b16	b16.a1	PFCIRM129_06070	Enolase 1	CYT	G	45.9	−26.5	20	5	–	1.1	−41.5	25	7	–	1.7
b17	b17.a1	PFCIRM129_07235	Methylmalonyl-CoA mutase small subunit (Methylmalonyl-CoA mutase beta subunit) (MCB-beta)	CYT	I	69.5	−16.2	9	4	–	0.4	−59.4	26	9	–	1.2
b19	b19.a1	PFCIRM129_10180	Iron-sulfur protein	CYT	C	57.2	−26.1	18	6	–	1.1	−16.7	8	3	–	0.4
b20	b20.a1	PFCIRM129_08670	Cell-wall peptidases, NlpC/P60 family SEC protein	SEC	M	58.7	−51.6	22	8	–	1.7	−9.5	6	2	–	0.4
b21	b21.a1	PFCIRM129_09300	FAD-dependent pyridine nucleotide-disulphide oxidoreductase:4Fe-4S ferredoxin, iron-sulfur binding:Aromatic-ring hydroxylase	CYT	C	59.7	–	–	–	–	–	−42.1	20	8	–	1.2
b22	b22.a1	PFCIRM129_00205	Succinate dehydrogenase flavoprotein subunit	CYT	C	74.7	−17.1	5	3	–	0.3	−20.8	6	4	–	0.5
b23	b23.a1	PFCIRM129_08495	NADH-quinone oxidoreductase chain G (NADH dehydrogenase I, chain G)	CYT	C	84.8	−22.3	6	3	–	0.2	−28.3	9	5	–	0.4
b24	b24.a1	PFCIRM129_09980	Peptidyl-prolyl cis-trans isomerase	SEC	O	35.9	−23.0	22	4	–	5.8	−11.5	7	2	–	1.2
b25	b25.a1	PFCIRM129_10295	Methylmalonyl-CoA carboxytransferase 12S subunit (EC2.1.3.1) (Transcarboxylase 12S subunit). 610 bp	CYT	I	56.3	−31.2	11	5	–	0.7	−15.2	7	3	–	0.4
b26	b26.a1	PFCIRM129_11300	Glyceraldehyde-3-phosphate dehydrogenase / erythrose 4 phosphate dehydrogenase	CYT	G	37.7	−48.7	39	9	–	2.9	–	–	–	–	–
b27	b27.a1	PFCIRM129_05155	ATP synthase subunit alpha (ATPase subunit alpha) (ATP synthase F1 sector subunit alpha)	CYT	C	58.8	−7.9	5	2	–	0.2	−17.9	11	5	–	0.6
b30	b30.a1	PFREUD_10490	ATP synthase subunit beta (ATPase subunit beta) (ATP synthase F1 sector subunit beta)	CYT	C	52.4	−12.0	9	3	–	0.4	−15.0	12	4	–	0.7
b31	b31.a1	PFCIRM129_11080 & PFCIRM129_11085	MERGED = TRUE	–	–	35.4	–	–	–	–	–	−17.1	20	4	–	0.9
b32	b32.a1	PFCIRM129_10995	Glycerol kinase (ATP:glycerol 3-phosphotransferase) (Glycerokinase) (GK)	CYT	C	55.6	–	–	–	–	–	−17.7	11	5	–	1.3
b33	b33.a1	PFCIRM129_01440	Coenzyme A transferase (Putative succinyl-CoA or butyryl-CoA:coenzyme A transferase)	CYT	C	55.6	–	–	–	–	–	−14.5	7	3	–	0.5
b34	b34.a1	PFCIRM129_11710 & PFCIRM129_11715	MERGED = TRUE	–	–	58.8	−12.1	4	2	–	0.4	−30.6	13	5	–	1.2
b35	b35.a1	PFCIRM129_05730	D-lactate dehydrogenase	CYT	C	63.6	−9.5	9	3	–	0.3	−14.4	11	4	–	0.4
b36	b36.a1	PFCIRM129_00390	Cysteine synthase 2	CYT	E	33.5	−40.2	38	6	–	1.7	–	–	–	–	–
b37	b37.a1	PFCIRM129_08120	Solute binding protein of the ABC transport system	SEC	E	61.4	−7.3	7	3	–	0.4	−11.9	4	2	–	0.3
b38	b38.a1	PFCIRM129_05105	Hypothetical protein	CYT	–	64	–	–	–	–	–	−17.0	10	5	–	0.6
b40	b40.a1	PFCIRM129_01500	Pyruvate phosphate dikinase	CYT	G	95.7	–	–	—-	–	–	−11.2	3	2	–	0.1
b41	b41.a1	PFCIRM129_03550	Alanine dehydrogenase	CYT	E	39.3	–	–	–	–	–	−5.8	6	2	–	0.4
b43	b43.a1	PFCIRM129_10420	iolA (Myo-inositol catabolism IolA protein) (Methylmalonic acid semialdehyde dehydrogenase)	CYT	C	52.7	–	–	–	–	–	−9.1	6	2	–	0.3
b44	b44.a1	PFCIRM129_08025	Resuscitation-promoting factor	SEC	L	37.7	−15.9	11	2	–	0.9	–	–	–	–	–
b45	b45.a1	PFREUD_14570	Polyribonucleotide nucleotidyltransferase (Polynucleotide phosphorylase) (PNPase) (Guanosine pentaphosphate synthetase)	CYT	J	79.3	–	–	–	–	–	−9.5	3	2	–	0.2
b46	b46.a1	PFCIRM129_08280	Elongation factor G (EF-G)	CYT	J	76.5	–	–	–	–	–	−5.4	2	2	–	0.2
b48	b48.a1	PFCIRM129_08935	FAD linked oxidase domain protein	CYT	C	100.4	–	–	–	–	–	−18.2	5	3	–	0.2
b49	b49.a1	PFCIRM129_08300	DNA-directed RNA polymerase beta chain (RNAP beta subunit) (Transcriptase beta chain) (RNA polymerase subunit beta)	CYT	K	128.5	–	–	–	–	–	−8.8	3	2	–	0.1
b50	b50.a1	PFCIRM129_00200	Succinate dehydrogenase	CYT	C	27	−8.4	10	2	–	0.6	–	–	–	–	–
b51	b51.a1	PFCIRM129_10175	Hypothetical protein	CYT	S	23.1	−11.5	16	2	–	0.7	–	–	–	–	–

a *The Group to which the protein belongs. All the proteins in a group have at least one peptide in common*.

b *The Sub-Group to which the protein belongs. All the proteins in a sub-group are identified with the same valid peptides*.

c *Protein description as it appears in the header of the fasta file*.

d *SurfG+ localization prediction*.

e *Cluster of Orthologous Group category – A, RNA processing and modification; B, Chromatin Structure and dynamics; C, Energy production and conversion; D, Cell cycle control and mitosis; E, Amino Acid metabolis and transport; F, Nucleotide metabolism and transport; G, Carbohydrate metabolism and transport; H, Coenzyme metabolis; I, Lipid metabolism; J, Translation; K, Transcription; L, Replication and repair; M, Cell wall/membrane/envelope biogenesis; N, Cell motility; O, Post-translational modification; P, Inorganic ion transport and metabolism; Q, Secondary Structure; T, Signal Transduction; U, Intracellular trafficking and secretion; Y, Nuclear structure; Z, Cytoskeleton; R, General Functional Prediction only; S, Function Unknown*.

f *Molecular weight of the protein expressed in KDa*.

g *Protein e-value expressed in log. Statistical value representing the number of times this protein would be identified randomly. Calculated as the product of unique peptide e-values in the sample*.

h *Percentage of protein sequence covered by identified peptides*.

i *The number of unique peptide sequence assigned to this protein*.

j *The number of unique peptide sequence specific to this subgroup of proteins. It is only available if there are more than one subgroup within a group*.

k *The Exponentially Modified Protein Abundance Index (emPAI) computation (Ishihama et al., 2005)*.

l *Part of these results were previously published in do Carmo et al. (2017)*.

### Impact of *slpB* mutation on *P. freudenreichii* ζ-potential and cell surface hydrophobicity

**Figure 1:**
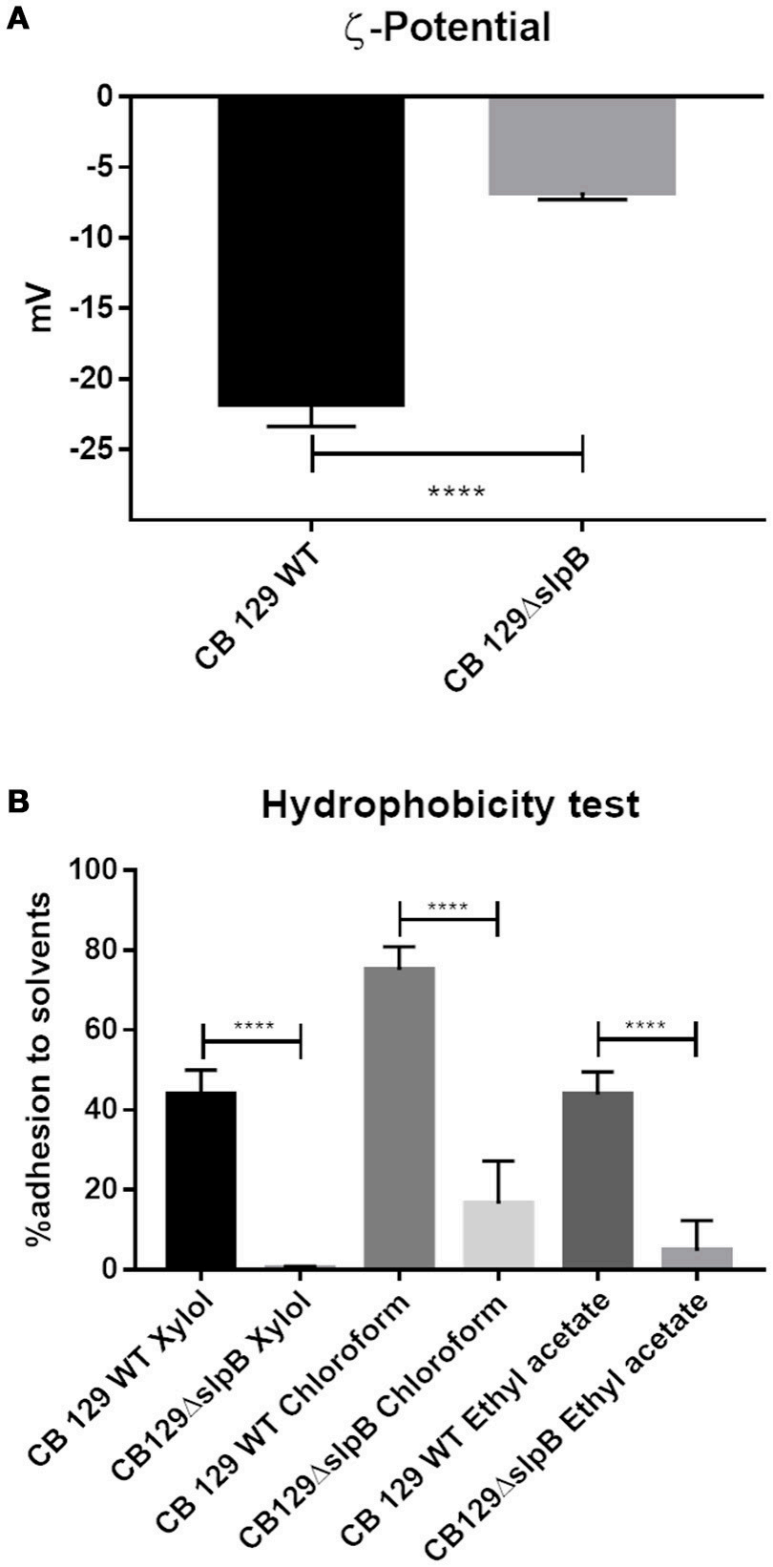
Mutation of *slp*B gene drastically affects surface proprieties in *P. freudenreichii* CIRM-BIA 129. **(A)** The surface net charge was determined by measuring the ζ-potential. **(B)** The surface hydrophobicity was determined by quantifying adhesion to solvents (Xylol, Chloroform, and Ethyl Acetate). Wild-type (WT) and mutant CB129Δ*slpB* strains were compared. Bar represents the mean SD of three biological replicates and three technical replicates. The asterisks (****) denotes the statistical significance of the represented value between CB 129 WT and CB 129Δ*slpB* (*p* > 0.0001).

**Figure 2:**
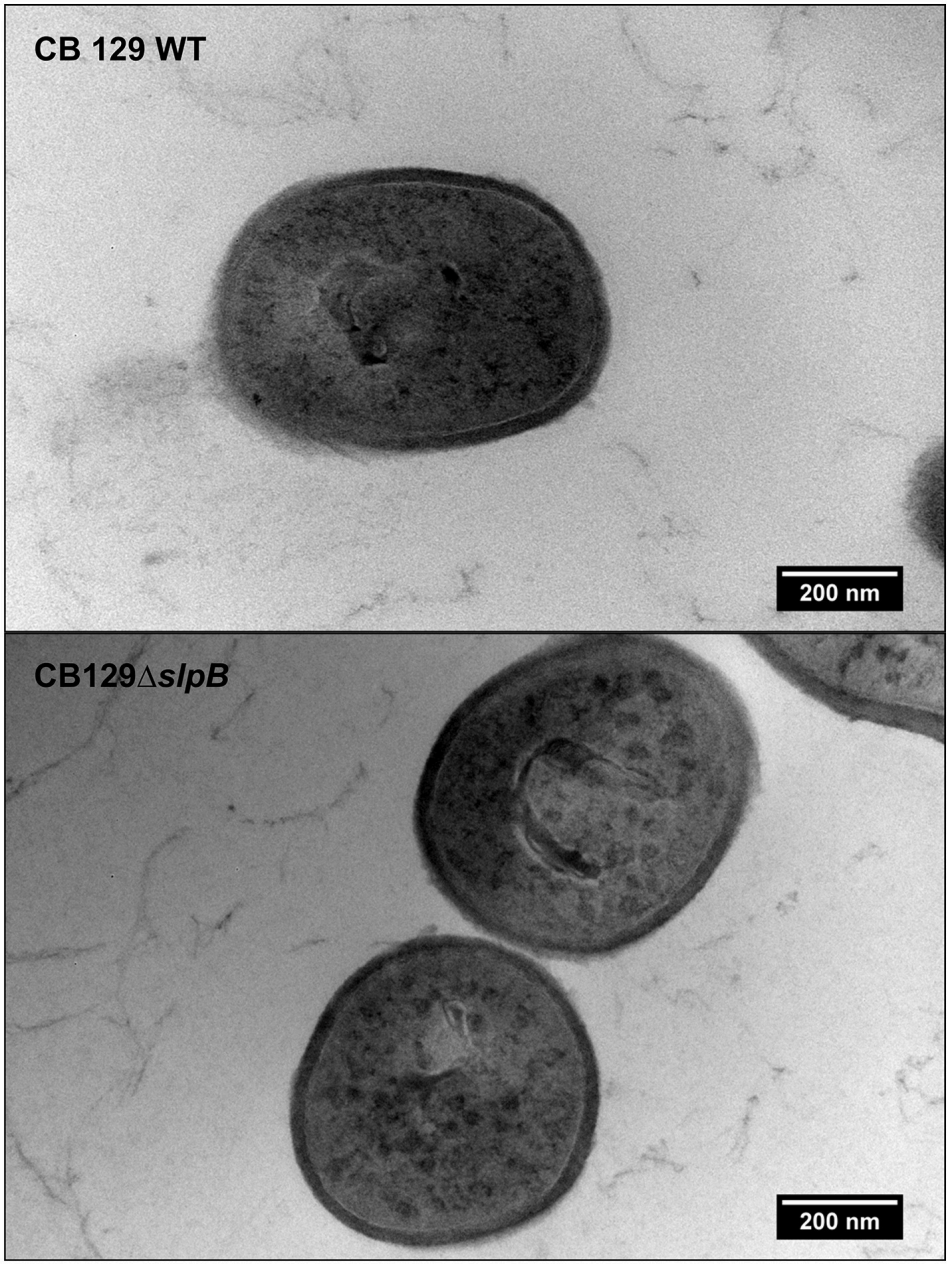
Mutation of *slp*B gene does not affect envelope thickness in *P. freudenreichii* CIRM-BIA 129. Wild-type (WT) and mutant CB129Δ*slpB* strains were analyzed by transmission electron microscopy (TEM). No difference in morphology and cell wall tickeness was found.

Propionibacterial SLPs, with a low isoelectric point, confer negative charges to the cell surface. In order to identify if the net surface charge was altered in the mutant strain, we conducted ζ-potential and cell surface hydrophobicity assays in both strains. As shown in the Figure 1A, the WT strain exhibited a zeta potential of −21.73 ± 1.63 mV, reflecting a negative net charge, in accordance with the low isoelectric point of *P. freudenreichii* SlpB protein. By contrast, mutation of *slpB* gene significantly affected the zeta potential of the CB129Δ*slpB* strain, which was −6.75 ± 0.55 mV, showing a reduced electronegativity, in accordance with a disorganization of the S-layer at the bacterial cell surface. As shown in Figure 1B, the wild type strain also showed a high affinity to the hydrocarbons tested, whereas the CB129Δ*slpB* mutant showed a decreased adhesion, whatever the hydrocarbon used in the assay. Adhesion, respectively to mutant and WT strains, were as follow: to Xylol, 0.33 ± 0.52% and 43.67 ± 6.31%, to Chloroform 16.5 ± 10.7% and 75 ± 5.88, and to Ethyl Acetate 5.33 ± 7.17% and 43.83 ± 5.74%. Cell surface properties being drastically affected, we then sought morphological changes caused by the mutation (Figure 2). Both strains exhibited a similar cell wall thickness, 24.33 ± 0.4154 nm and 24.90 ± 0.4154 nm, respectively. No significant difference in term of bacteria morphology, cell wall thickness and shape was observed between the two strains using transmission electron microscopy.

### Impact of *slpB* mutation on *P. freudenreichii* growth and stress tolerance

**Figure 3:**
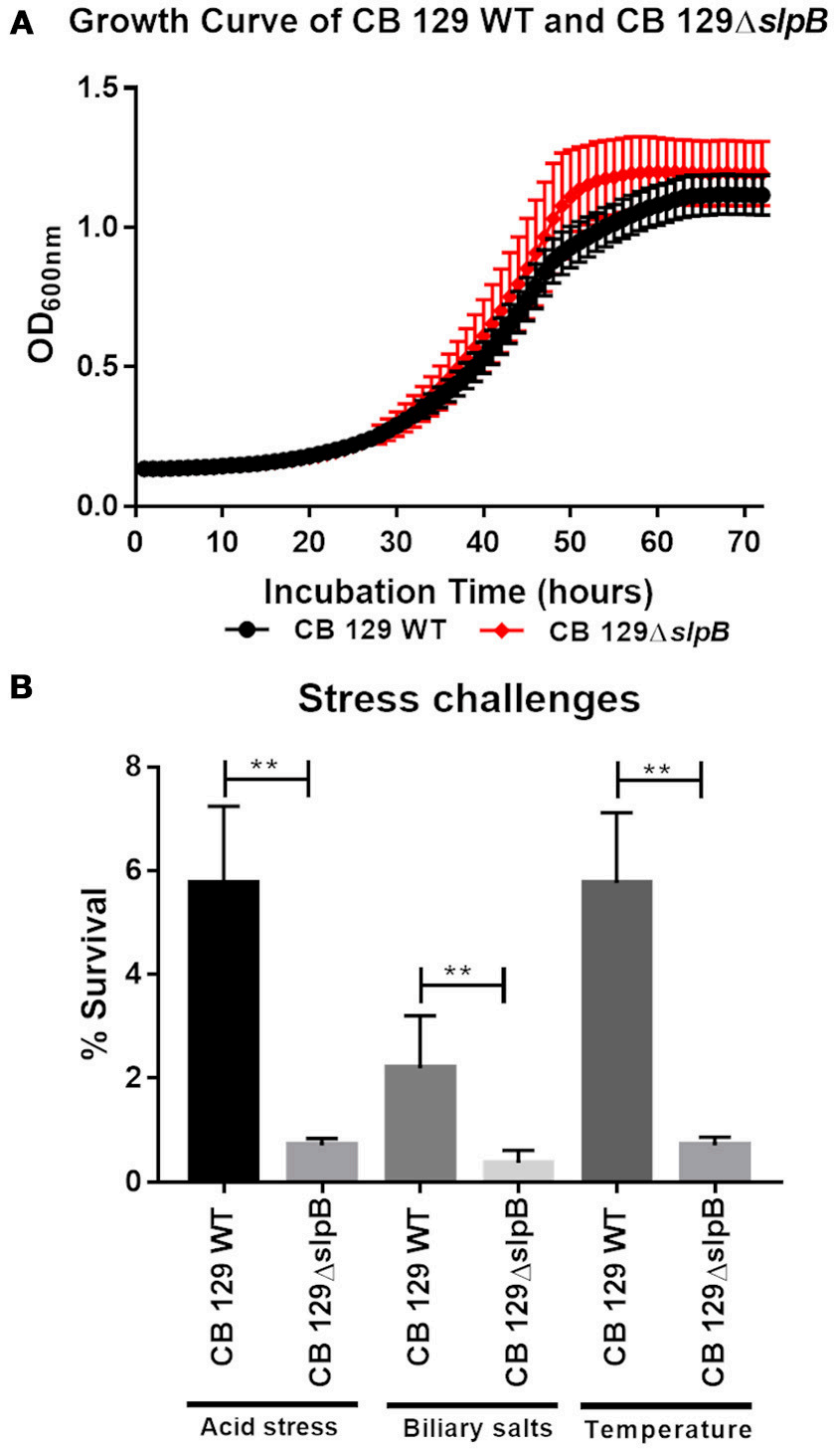
Mutation of *Slp*B drastically affects stress tolerance in *P. freudenreichii* CIRM-BIA 129. **(A)** The growth curve of Wild-type (WT) and mutant CB129Δ*slpB* strains was determined at 30°C in YEL broth until stationary phase (72 h). Growth was monitored by OD_650 nm_ as a function of time. No statistically significant difference was found in growth curve between strains. **(B)** Wild-type (WT) and mutant 129Δ*slpB* strains were subjected to acid, bile salts and thermal challenges. Viable propionibacteria were enumerated by plate counting before and after each challenge. Asterix represent statistically significant differences between strains and were indicated as follows: **p < 0.01.

A single mutation, inactivating a key gene, may affect bacterial fitness and thus probiotic efficacy. We therefore monitored *P. freudenreichii* growth and tolerance toward acid, bile salts and heat challenges, in the wild type and in the mutant. The growth curves showed a similar pattern for both strains (Figure 3A). The bacterial count at the stationary phase end was also equivalent for both strains, with a viable population count of 1.63 × 10^9^ CFU.mL^−1^ and 1.75 × 10^9^ CFU.mL^−1^ for the wild type and the mutant strains, respectively. Tolerance toward stress challenges is reported in Figure 3B. In the case of acid stress, we observed a significant decrease in viability for the CB129Δ*slpB* strain 0.71 ± 0.13% (7.3 × 10^6^ CFU.mL^−1^) compared to the WT strain 5.76 ± 1.48% (5.76 × 10^7^ CFU.mL^−1^). During the bile salts stress, we observed the same trend in the tolerance. Indeed, the survival rate for the CB129Δ*slpB* strain was significantly decreased 0.37 ± 0.24% (3.71 × 10^6^ CFU.mL^−1^), compared to the WT strain 2.19 ± 1.01% (2.19 × 10^7^ CFU.mL^−1^). The same stands for heat challenge, with a reduced survival in CB129Δ*slpB* 0.71 ± 0.16% (9.01 × 10^6^ CFU.mL^−1^) compared to WT strain 5.76 ± 1.35% (5.86 × 10^7^ CFU.mL^−1^).

### Impact of *slpB* mutation on *P. freudenreichii* qualitative and quantitative proteome

**Figure 4:**
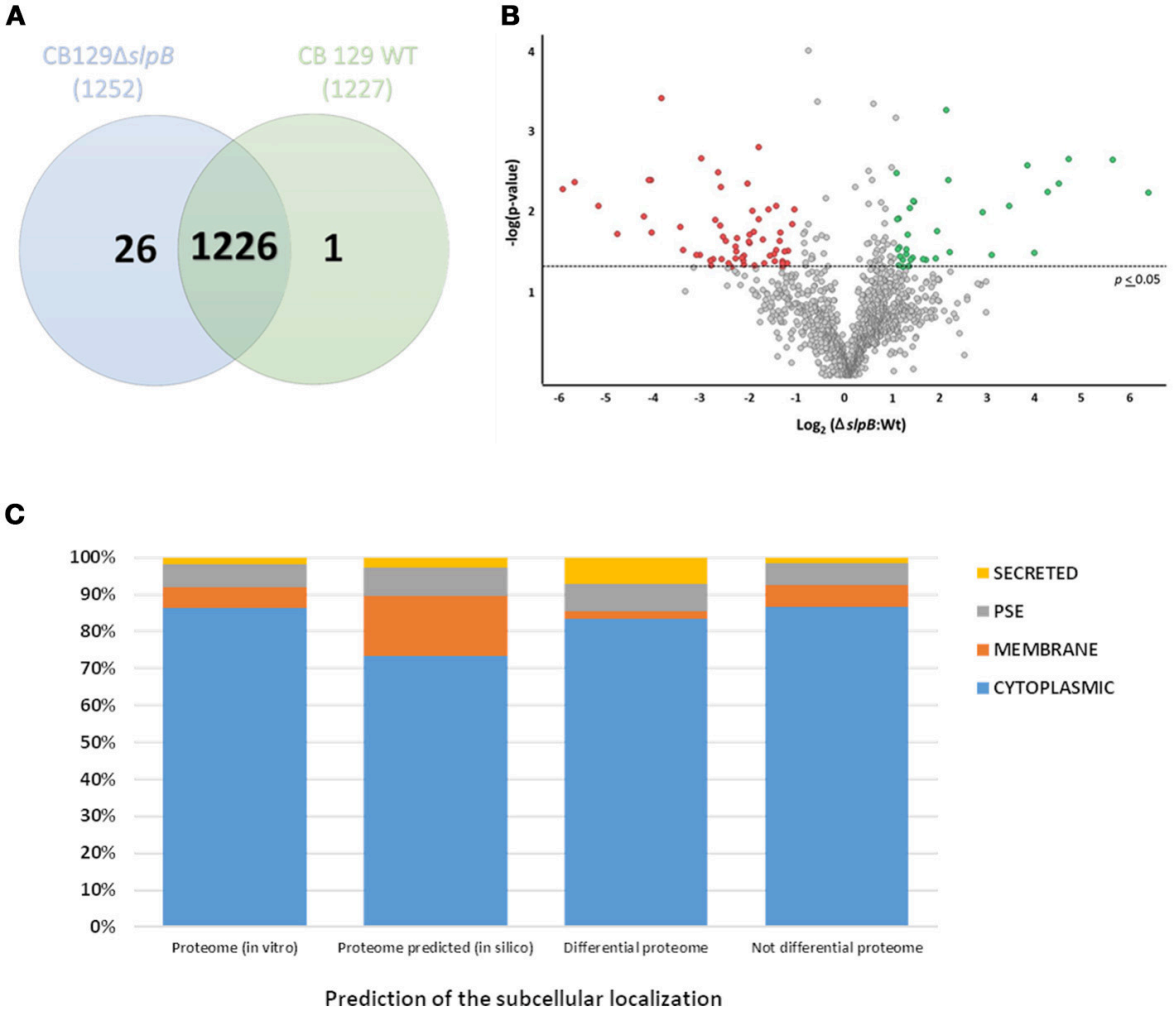
Label-free quantification of proteins from *P. freudenreichii* CIRM-BIA 129 and CB 129Δ*slpB* strain. **(A)** Distribution of the proteins identified in the proteome of WT and CB129Δ*slpB* strains, represented by a Venn diagram. **(B)** Volcano Plot showing Log(2) Fold Change of the differentially expressed proteins in CB129Δ*slpB* strain in relation to WT strain. Green (up-regulated proteins) and red circles (down-regulated proteins) represent proteins statistically different (*p* ≤ 0.05, ANOVA) in abundance between strains by 2-fold or more. **(C)** Prediction of the subcellular localization of the proteins identified by LC/MS and organized as cytoplasmic (CYT), membrane (MEM), potentially surface-exposed (PSE) or secreted proteins (SEC).

Considering the major alterations in surface extractable proteins, bacteria cell surface physicochemical properties, and stress tolerance, a qualitative and quantitative analysis of the total proteome was performed to elucidate the impact of the *slpB* gene knockout in the mutant strain. A total of 1,288 quantifiable proteins (53.26% of predicted proteome) wherein 1,253 proteins (reported in Figure 4A) were identified (Table S1). In the WT strain 1,227 proteins were found, whereas in the CB129Δ*slpB* strain, we detected 1,252 proteins. Comparative analysis revealed a core-proteome, composed by 1,226 proteins, shared by both strains (Figure 4A). Differences in protein abundance were observed by proteomic quantitative analysis (Figure 4B). A total of 97 proteins (4.2% of the predicted proteome) of these common proteins showed differences in the level of expression among strains, including 36 up-regulated and 61 down-regulated proteins in CB129Δ*slpB* in comparison with the WT strain (Table 2).

**Table 2:** Differentially regulated proteins at CB129Δ*slpB* in relation to CB 129 wild-type.

**Accession**	**Score**	**Description**	**LOG(2) ratio fold-change**	**Anova (p)**	**COG biological process**
**UP-REGULATED PROTEINS**					
PFCIRM129_09610	41.9018	Protein of unknown function	6.16	0.006	Coenzyme transport and metabolism and Signal transduction mechanisms
PFCIRM129_09540	37.0751	Protein of unknown function	5.43	0.003	Transcription
PFCIRM129_09590	102.7882	Protein of unknown function	4.53	0.002	Cell wall/membrane/envelope biogenesis
PFCIRM129_09465	51.2086	Protein of unknown function	4.33	0.005	–
PFCIRM129_09585	90.2656	Protein of unknown function	4.10	0.006	General function prediction only
PFCIRM129_04060	38.8837	Guanylate kinase, Guanosine monophosphate kinase (GMP kinase)	3.83	0.033	Nucleotide transport and metabolism
PFCIRM129_09570	44.4682	Protein of unknown function	3.69	0.003	Cell motility
PFCIRM129_07005	243.5985	DNA ligase (NAD+)	3.32	0.009	Replication, recombination and repair
PFCIRM129_01620	59.7705	Stomatin/prohibitin	2.96	0.036	Posttranslational modification, protein turnover, chaperones
PFCIRM129_10485	35.443	Spermidine synthase	2.76	0.011	Amino acid transport and metabolism
PFCIRM129_10870	30.3436	Protein of unknown function	2.09	0.033	General function prediction only
PFCIRM129_09930	56.3355	Hypothetical protein	2.06	0.004	Posttranslational modification, protein turnover, chaperones
PFCIRM129_09935	87.1427	Aldo/keto reductase	2.02	0.001	Secondary metabolites biosynthesis, transport and catabolism
PFCIRM129_08225	203.0233	50S ribosomal protein L2	1.84	0.018	Translation, ribosomal structure and biogenesis
PFCIRM129_05110	60.8023	Nuclease of the RecB family	1.80	0.039	Replication, recombination and repair
PFCIRM129_02560	52.268	Transcriptional regulator	1.61	0.041	Coenzyme transport and metabolism
PFCIRM129_08430	75.8136	Pyruvate flavodoxin/ferredoxin oxidoreductase	1.55	0.040	Energy production and conversion
PFCIRM129_09920	380.2718	Hypothetical secreted protein	1.37	0.008	Translation, ribosomal structure and biogenesis
PFCIRM129_04715	57.0906	Hypothetical protein	1.36	0.008	Signal transduction mechanisms
PFCIRM129_09175	100.5874	NAD-dependent epimerase/dehydratase	1.33	0.038	General function prediction only
PFCIRM129_12405	136.3375	UDP-glucose 4-epimerase	1.29	0.041	Cell wall/membrane/envelope biogenesis
PFCIRM129_01790	34.8378	3-dehydroquinate dehydratase	1.27	0.010	Amino acid transport and metabolism
PFCIRM129_07890	128.6333	Putative O-sialoglycoprotein endopeptidase	1.26	0.048	Translation, ribosomal structure and biogenesis
PFCIRM129_00585	212.5378	Polyphosphate glucokinase	1.24	0.048	Transcription and Carbohydrate transport and metabolism
PFCIRM129_07790	140.7785	Cysteine synthase 1	1.23	0.020	Amino acid transport and metabolism
PFCIRM129_09600	51.9916	Protein of unknown function	1.21	0.034	Replication, recombination and repair
PFCIRM129_11300	522.5826	Glyceraldehyde-3-phosphate dehydrogenase/erythrose 4 phosphate dehydrogenase	1.21	0.030	Carbohydrate transport and metabolism
PFCIRM129_00690	23.8969	Protein of unknown function	1.14	0.049	Function unknown
PFCIRM129_01510	22.4775	Carbohydrate or pyrimidine kinases PfkB family	1.14	0.040	Carbohydrate transport and metabolism
PFCIRM129_03870	27.4108	Glutamine-dependent NAD(+) synthetase	1.08	0.036	General function prediction only
PFCIRM129_00225	85.9906	16S rRNA processing protein	1.06	0.028	Translation, ribosomal structure and biogenesis
PFCIRM129_11255	221.963	Pyridoxal biosynthesis lyase pdxS	1.06	0.047	Coenzyme transport and metabolism
PFCIRM129_03920	293.1815	Pyridine nucleotide-disulphide oxidoreductase	1.05	0.013	Energy production and conversion
PFCIRM129_07930	409.5736	Glucosamine–fructose-6-phosphate aminotransferase (Hexosephosphate aminotransferase, D-fructose-6-phosphate amidotransferase)	1.04	0.031	Cell wall/membrane/envelope biogenesis
PFCIRM129_11805	158.7382	Magnesium (Mg2+) transporter	1.03	0.013	Inorganic ion transport and metabolism
PFCIRM129_08045	417.9752	DNA-directed RNA polymerase alpha chain (RNAP alpha subunit) (Transcriptase alpha chain) (RNA polymerase subunit alpha)	1.01	0.004	Transcription
**DOWN-REGULATED PROTEINS**					
PFCIRM129_06035	66.5616	Enolase 2	−1.09	0.010	Carbohydrate transport and metabolism
PFCIRM129_06325	41.3227	Trypsin-like serine protease	−1.13	0.015	Posttranslational modification, protein turnover, chaperones
PFCIRM129_00315	221.1159	Beta-lactamase-like:RNA-metabolizing metallo-beta-lactamase	−1.21	0.031	Translation, ribosomal structure and biogenesis
PFCIRM129_04530	19.2011	Hypothetical protein	−1.23	0.045	Function unknown
PFCIRM129_06605	17.9867	Metal-dependent hydrolase	−1.29	0.032	General function prediction only
PFCIRM129_10030	162.9893	DNA repair protein	−1.32	0.042	Replication, recombination and repair
PFCIRM129_06500	87.9342	Hypothetical protein	−1.33	0.048	Nucleotide transport and metabolism
PFCIRM129_10650	33.3856	Hypothetical protein	−1.33	0.045	Cell wall/membrane/envelope biogenesis
PFCIRM129_03835	79.2532	Pyrazinamidase/nicotinamidase	−1.37	0.019	Coenzyme transport and metabolism and Signal transduction mechanisms
PFCIRM129_10070	83.9023	Hypothetical protein	−1.39	0.024	General function prediction only
PFCIRM129_00245	381.851	GTP binding signal recognition particle protein	−1.45	0.031	Intracellular trafficking, secretion, and vesicular transport
PFCIRM129_05955	85.1759	Peptide-methionine (S)-S-oxide reductase	−1.46	0.009	Posttranslational modification, protein turnover, chaperones
PFCIRM129_09830	327.7897	Aspartyl/glutamyl-tRNA(Asn/Gln) amidotransferase subunit B (Asp/Glu-ADT subunit B)	−1.49	0.042	Translation, ribosomal structure and biogenesis
PFCIRM129_09395	77.9918	Protein of unknown function	−1.50	0.035	Replication, recombination and repair
PFCIRM129_07355	38.1315	Hypothetical protein	−1.59	0.036	Amino acid transport and metabolism
PFCIRM129_02750	19.5802	Anti-sigma factor	−1.61	0.010	Transcription
PFCIRM129_09840	37.8042	Glutamyl-tRNA(Gln) amidotransferase subunit C (Aspartyl/glutamyl-tRNA(Asn/Gln) amidotransferase subunit C)	−1.71	0.045	Translation, ribosomal structure and biogenesis
PFCIRM129_12290	113.782	Hypothetical protein	−1.73	0.023	Translation, ribosomal structure and biogenesis
PFCIRM129_02880	157.3643	Zn dependant peptidase	−1.82	0.002	General function prediction only
PFCIRM129_01675	171.214	Flavin-containing amine oxidase	−1.82	0.013	Amino acid transport and metabolism
PFCIRM129_00465	78.5201	Thiamine biosynthesis protein	−1.90	0.047	Coenzyme transport and metabolism
PFCIRM129_09980	56.8929	Peptidyl-prolyl cis-trans isomerase	−1.91	0.019	Posttranslational modification, protein turnover, chaperones
PFCIRM129_02370	174.428	L-aspartate oxidase (LASPO) (Quinolinate synthetase B)	−1.94	0.010	Coenzyme transport and metabolism
PFCIRM129_05120	33.1399	Putative carboxylic ester hydrolase	−1.99	0.020	Lipid transport and metabolism
PFCIRM129_04475	54.968	Transporter	−2.01	0.026	Function unknown
PFCIRM129_12425	80.5954	Protein of unknown function FUZZYLOCATION = TRUE	−2.02	0.025	Transcription
PFCIRM129_04980	227.0852	D-alanine–D-alanine ligase (D-alanylalanine synthetase)	−2.05	0.005	Cell wall/membrane/envelope biogenesis and General function prediction only
PFCIRM129_11215	88.6965	Dioxygenase	−2.12	0.047	Inorganic ion transport and metabolism and Secondary metabolites biosynthesis, transport and catabolism
PFCIRM129_10195	96.8755	Transcriptional regulator	−2.12	0.036	Transcription
PFCIRM129_08985	30.4822	Hypothetical protein	−2.13	0.042	General function prediction only
PFCIRM129_04260	287.3443	DNA polymerase III alpha subunit	−2.15	0.038	Replication, recombination and repair
PFCIRM129_02065	15.789	Ferrous iron uptake protein A 9.a.8.1.x	−2.25	0.022	Inorganic ion transport and metabolism
PFCIRM129_04725	106.5589	Hypothetical protein	−2.27	0.032	Cell wall/membrane/envelope biogenesis
PFCIRM129_05460	489.2107	Surface protein with SLH domain	−2.29	0.039	Posttranslational modification, protein turnover, chaperones
PFCIRM129_04925	12.884	Hypothetical protein	−2.29	0.028	Carbohydrate transport and metabolism
PFCIRM129_10690	9.166	Protein of unknown function	−2.37	0.049	Function unknown
PFCIRM129_05620	65.4691	MscS transporter, small conductance mechanosensitive ion channel	−2.43	0.044	Cell wall/membrane/envelope biogenesis
PFCIRM129_06895	73.9719	Thiredoxine like membrane protein	−2.49	0.024	Posttranslational modification, protein turnover, chaperones
PFCIRM129_10610	181.5134	Phosphocarrier, HPr family	−2.54	0.021	Signal transduction mechanisms and Carbohydrate transport and metabolism
PFCIRM129_02565	36.2455	Hypothetical protein	−2.57	0.039	Defense mechanisms
PFCIRM129_00850	58.5524	Cation-transporting ATPase	−2.59	0.005	Inorganic ion transport and metabolism
PFCIRM129_02970	142.4983	Hypothetical protein	−2.60	0.016	Energy production and conversion
PFCIRM129_00010	145.5914	Argininosuccinate lyase (Arginosuccinase)	−2.65	0.004	Amino acid transport and metabolism
PFCIRM129_02590	36.8971	Hypothetical transmembrane protein	−2.71	0.013	Inorganic ion transport and metabolism
PFCIRM129_02910	44.2268	Hypothetical protein	−2.74	0.039	Replication, recombination and repair
PFCIRM129_10040	39.9232	Hypothetical protein	−2.78	0.048	Carbohydrate transport and metabolism
PFCIRM129_12235	1098.1026	Internaline A	−2.80	0.041	Posttranslational modification, protein turnover, chaperones
PFCIRM129_00040	20.5108	N-acetyl-gamma-glutamyl-phosphate reductase (AGPR) (N- acetyl-glutamate semialdehyde dehydrogenase) (NAGSA dehydrogenase)	−2.99	0.002	Amino acid transport and metabolism
PFCIRM129_03005	41.8204	Hypothetical protein	−3.01	0.035	Secondary metabolites biosynthesis, transport and catabolism
PFCIRM129_05445	69.2875	Transcriptional Regulator, TetR family	−3.09	0.036	Transcription
PFCIRM129_02960	83.486	Cold shock-like protein CspA	−3.36	0.031	Transcription
PFCIRM129_00705	46.3689	Surface protein of unknown function	−3.42	0.016	–
PFCIRM129_08670	192.0452	Cell-wall peptidases, NlpC/P60 family secreted protein	−3.80	0.000	General function prediction only
PFCIRM129_03390	45.4963	Superfamily II RNA helicase	−4.01	0.019	Replication, recombination and repair
PFCIRM129_06155	35.4	Hypothetical protein	−4.03	0.004	Carbohydrate transport and metabolism
PFCIRM129_06085	371.7356	Transcription-repair coupling factor	−4.07	0.004	Replication, recombination and repair and Transcription
PFCIRM129_01360	47.9803	NUDIX hydrolase	−4.17	0.012	Nucleotide transport and metabolism
PFCIRM129_11775	48.0011	Surface protein D with SLH domain	−4.70	0.020	Posttranslational modification, protein turnover, chaperones
PFCIRM129_00700	461.2371	Surface layer protein B (S-layer protein B)	−5.10	0.009	Posttranslational modification, protein turnover, chaperones
PFCIRM129_11140	154.2908	Type I restriction-modification system DNA methylase	−5.58	0.005	Defense mechanisms
PFCIRM129_04135	15.2209	Uncharacterized ATPase related to the helicase subunit of the holliday junction resolvase	−5.82	0.006	Replication, recombination and repair

According to the predicted subcellular localization of the 1,253 proteins identified, 1,081 proteins are CYT (61% of predicted proteome), 71 are MEM (18% of predicted proteome), 77 are PSE (41% of predicted proteome) and 24 are SEC (38% of predicted proteome). In the analysis of non-differentially expressed proteins, we classified 1,001 as CYT proteins, 67 as MEM proteins, 70 as PSE proteins and 22 as SEC proteins (Figure 4C). Meanwhile, between the *P. freudenreichii* WT and the CB129Δ*slpB* strains, from 97 proteins differentially expressed, the subcellular localization were predicted as follow: 81 CYT, 2 MEM, 7 PSE, and 7 SEC proteins (Figure 4C).

**Figure 5:**
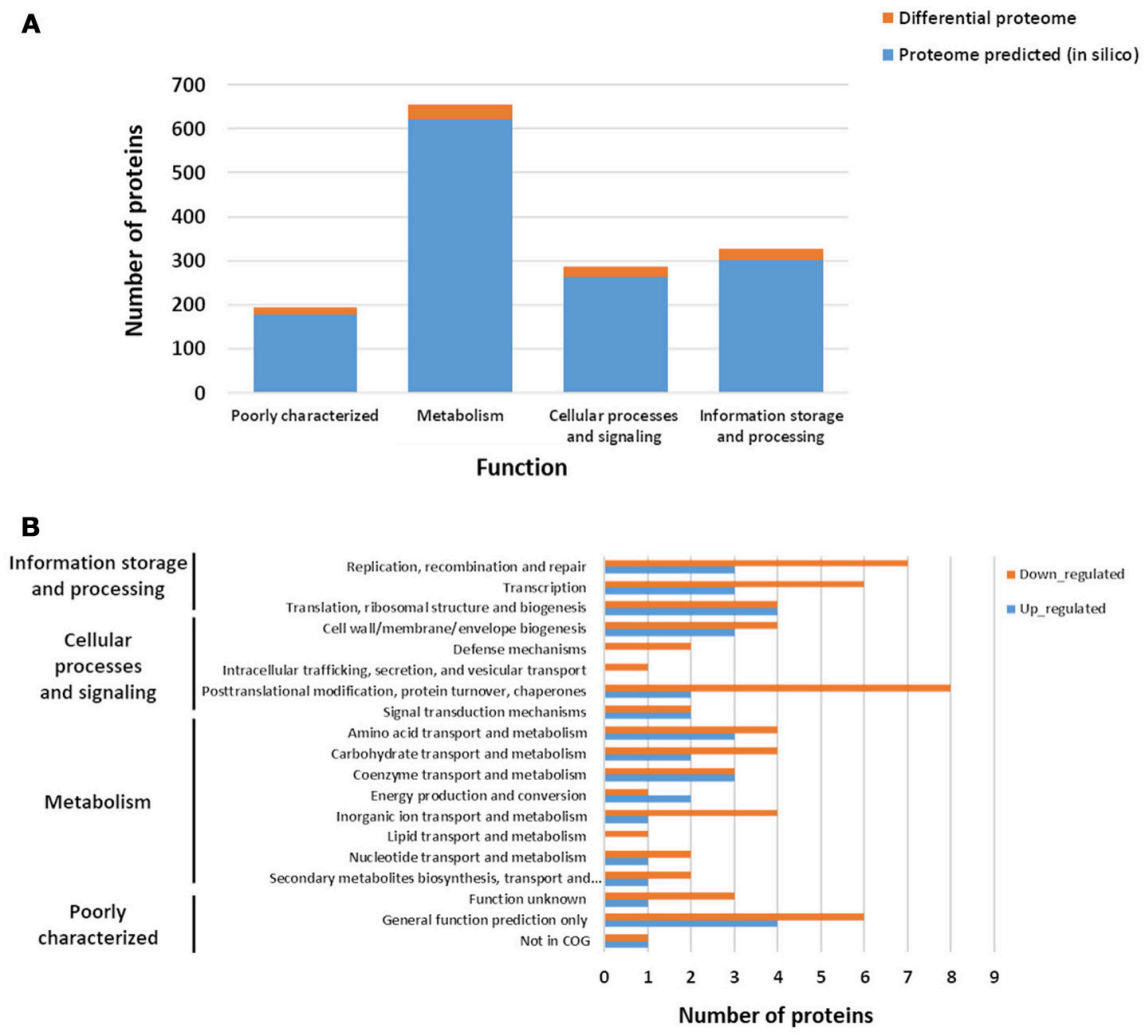
Repartition of differential proteins in biological processes. **(A)** Functional distribution of the predicted theoretical proteome and of the experimental differential proteins when comparing Wild-type (WT) and mutant CB129Δ*slpB* strains. **(B)** Repartition of differential proteins in Biological processes. Functional distribution and Biological process were predicted based on the functional classifications of COG database.

According to COG functional classifications, the differentially expressed proteins were classified into 20 biological processes (Figure 5A). A general category of differentially regulated proteins in CB129Δ*slpB* strain core proteome showed 27 proteins involved in information storage and processing, 25 associated to metabolism and, 18 proteins related to cellular processes and signaling (Figure 5A). Proteins that mediate different biological process were dysregulated in the mutant strain. As seen in Figure 5B, 11 proteins were classified as having general functions, 10 proteins related to process of replication, recombination and repair, other 10 proteins linked to posttranslational modification, chaperones, protein turnover, and 9 proteins involved in the transcription process. The differentially expressed proteins between wild-type and mutant strains detected in each functional category are shown in Table 2. In addition, we detected proteins exclusive to the proteome of each strain. WT strain exhibits a unique exclusive protein, the Putative carboxylic ester hydrolase, which is involved in metabolism, especially in hydrolase activity. Interestingly, 27 proteins were found exclusively in the mutant strain, they are involved in several processes like metabolism and replication, recombination and repair (Table S1).

### *slpB* gene mutagenesis and whole-genome co-localization

**Figure 6:**
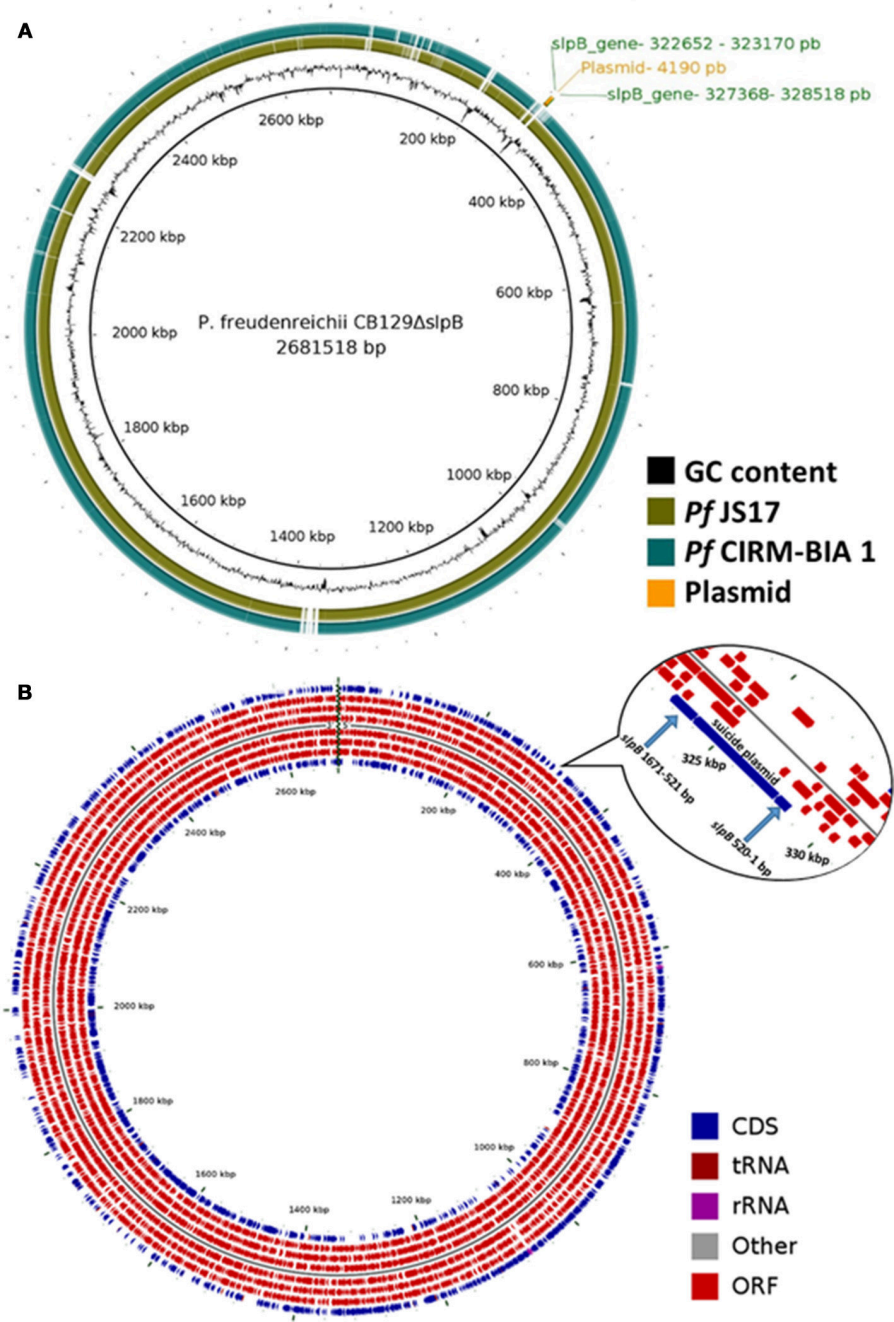
Comparative genomic map generated with BRIG and Map of Circular genome generated with CGview. **(A)** *P. freudenreichii* CIRM-BIA 1 and *P. freudenreichii* JS17 were aligned using CB129Δ*slpB* strain as a reference. **(B)** In the outermost ring the genes localization in genome, followed by CDS, tRNAs, rRNAs, other RNAs, and CDSs. The insertion site of the plasmid for the *slpB* gene mutation is visualized in the zoom image.

Complete genome of CB129Δ*slpB* (BioProject - PRJNA476583, Accession - CP030279) strain was sequenced and assembled in a circular chromosome, which exhibits a length of 2.6815.18 bp, with a G+C content of 67.28%, and a total of 2,479 CDSs, 6 rRNA genes (5S, 16S, and 23S), and 45 tRNA genes. The circular map showed a high similarity when comparing CB129Δ*slpB* with the CIRM-BIA 1 and the JS17 reference strains (Figure 6A). Figure 6B shows the localization of the plasmid inserted within the *slpB* gene during its knockout and Figures S1, S2 shows the read mapping before and after the insertion. Genomic analyses of genetic context, i.e., the sequences upstream and downstream the *slpB* gene, confirmed that this locus is not part of an operon and thus should not affect the expression of downstream genes or upstream genes. Complete genome sequence of CB129Δ*slpB* strain further ruled out any homologous recombination (HR) in other genome sites.

### Protein-protein interaction (PPI)

**Figure 7:**
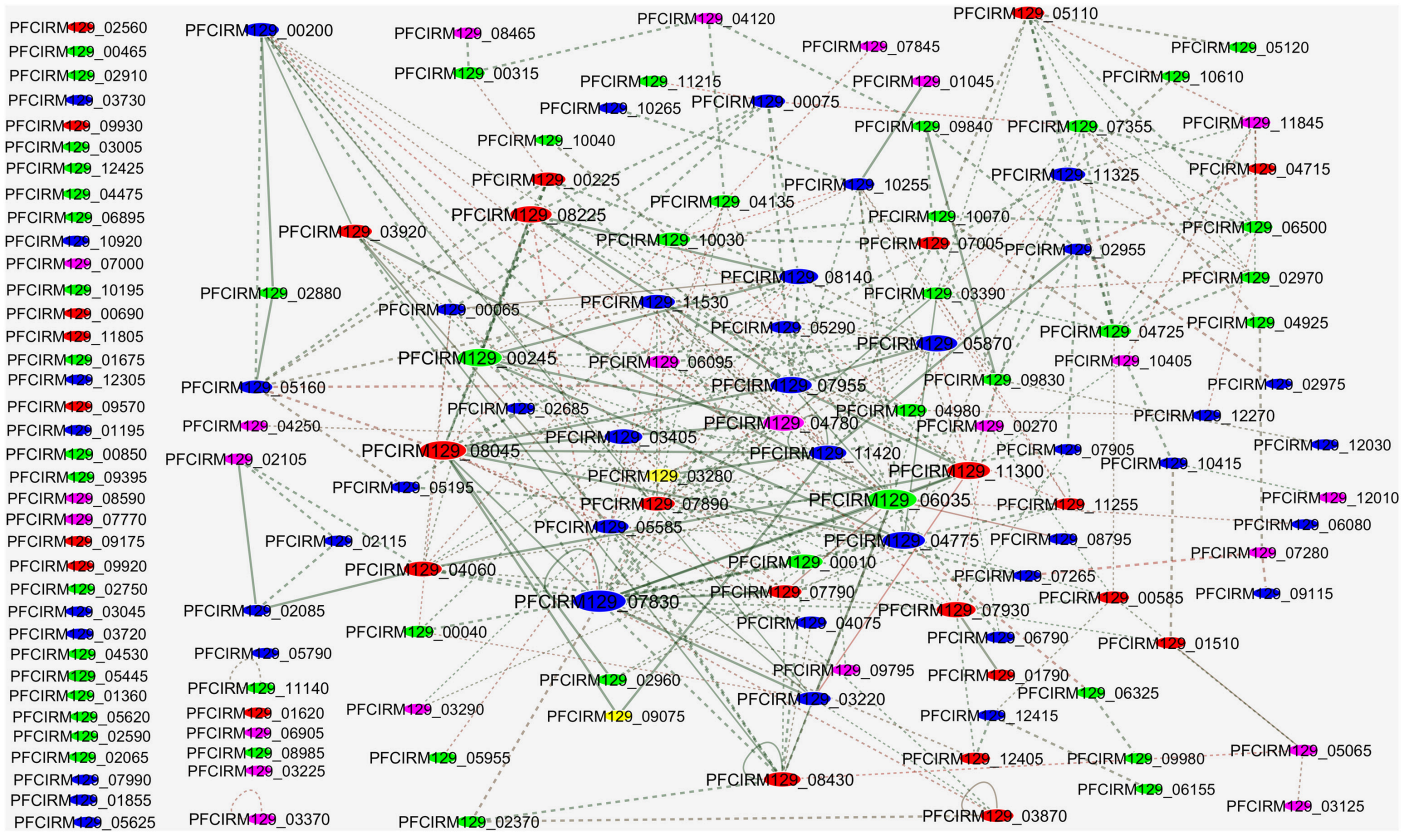
Protein-protein interactions of the proteins identified as differentially expressed in CB 129Δ*slpB*. The sizes of the nodes represent the degree of interaction for each gene/protein; the major nodes demonstrate greater interactions. Red, up-regulated; Blue, unchanged; Green, down-regulated; Yellow, Exclusive identified at WT strain; Purple, Exclusive identified at CB129Δ*slpB* strain.

We performed a PPI network to evaluate the interactions among the proteins differentially regulated in WT and CB129Δ*slpB* strains (Figure 7). The interactome analysis revealed 118 interactions between identified proteins. In PPI network, we observed that upregulated proteins, such as DNA-directed RNA polymerase alpha chain (PFCIRM129_08045), and 50S ribosomal protein L2 (PFCIRM129_08225), which exhibit high interaction, are involved in Transcription and Translation, respectively. Moreover, downregulated proteins such as GTP binding signal recognition particle protein (PFCIRM129_00245), DNA polymerase III alpha subunit (PFCIRM129_04260) and Enolase 2 (PFCIRM129_06035) showing high interaction, are involved in metabolism, DNA repair and main glycolytic pathway, respectively.

## Discussion

*Propionibacterium freudenreichii* CIRM-BIA 129 has emerged as a probiotic strain with a great immunomodulatory potential in the context of inflammatory bowel disease, according to promising results obtained in animal models (Plé et al., 2015, 2016). Recently, our group has studied the role of the surface SlpB protein of *P. freudenreichii* CIRM-BIA 129 in adhesion to the intestinal epithelial cells, a probiotic property linked to beneficial effects. Knocking-out of the *slpB* gene evidenced a direct involvement of this protein in the adhesion to HT-29 cells. Electrophoretic analysis of guanidine extracts confirmed the disappearance of the corresponding SlpB protein (do Carmo et al., 2017). Surface layer proteins are associated to several functions (do Carmo et al., 2018). Therefore, in order to better understand the impact of this mutation, we performed a more thorough proteomic analysis by applying nanoLC-MS/MS to these extracts. Differences were found between the parental wild type CIRM BIA 129 and the isogenic CB129Δ*slpB* mutant strains of *P. freudenreichii*, in terms of surface extractable proteins. As shown in Table 1, proteins previously identified in CB 129 WT strain guanidine-extracted proteins (Le Maréchal et al., 2015) were detected in both strains, including in particular, surface proteins anchored in the peptidoglycan cell wall via surface layer homology (SLH) domains, such as SlpA, SlpB, SlpE, and InlA like as previously reported by Carmo and collaborators (do Carmo et al., 2017). However, this set of SLH domain-containing proteins was reduced in the mutant strain guanidine-extracted proteins, with the expected absence of SlpB protein, thus validating the directed mutagenesis. Analysis of CB129Δ*slpB* strain guanidine-extracted proteins, identified several proteins, including chaperones, such as ClpB, DnaK, and GroEL, and Enolase (carbohydrate metabolism) involved in stress tolerance, as previously reported for *Propionibacterium* ssp. strains by enzymatic shaving of the surface proteins using trypsin (Jan et al., 2000; Gagnaire et al., 2015; Huang et al., 2016). Another noticeable difference was the higher number of guanidine-extracted proteins, in the mutant strain, compared to the wild type strain. This included proteins usually described as cytoplasmic: enzymes of the central carbon metabolic pathways, such as pyruvate synthase, or the two subunits of the methylmalonyl-CoA mutase, a recognized cytoplasmic marker, previously described as an extracellular marker of autolysis (Valence et al., 2000). Interestingly, the HSP 70 cytoplasmic stress-related protein present at the surface of the mutant strain could be responsible for preventing protein denaturation. It is as such considered a factor of virulence and pathogenesis in some specific pathogens (Ghazaei, 2017), in *Neisseri meingitidis* (Knaust et al., 2007) and in *Mycobacterium* spp. (Das Gupta et al., 2008). This appeals further investigation, as it suggests a profound modification of the envelope structure and cell surface properties of the mutant strain.

SLAPs are known to determine key parameters of the surface layer of bacteria, in terms of charge and hydrophobicity (Wilson et al., 2001). Not only amino acid residues, but also covalent modification may endow the S-layer lattice with a strong negative charge. Thus, we determined the surface charge in both *P. freudenreichii* WT and CB129Δ*slpB* strains by measuring the zeta potential, which reflects the mobility rate of cells within an electric field. A lower negative value is reportedly linked with higher hydrophobicity, consequently improving adhesion (de Wouters et al., 2015). Likewise, considering the presence of surface proteins and their role in zeta potential, van der Mei *et al*. have shown that some wild type strains, like the *L. acidophilus* ATCC4356, with SLPs, are more negatively charged at pH 7 than strains without SLPs, such as *L. johnsonii* LMG9436 and *L. gasseri* LMG9203 (van der Mei et al., 2003). We thus further investigated the hydrophobicity of the cell surface, a parameter thought to be correlated with *in vitro* adhesion of bacteria to mucin, collagen, fibronectin, and to human epithelial cells (Duary et al., 2011). The cell surface hydrophobic and hydrophilic properties have been studied in lactic acid bacteria (Sandes et al., 2017) and can be correlated to the adhesion process to intestinal epithelial cells of apolar surface proteins (Guo et al., 2010). Using the MATH assay, we showed that the CB129Δ*slpB* strain has a strongly decreased ability to adhere to xylol, as well as to chloroform and to ethyl acetate solvents, indicating a change in the global properties of the cell surface, affecting adhesion to surfaces. These results corroborate with the previous study showing a decreased adhesion to HT-29 human intestinal epithelial cells (do Carmo et al., 2017). Hydrophobicity and ζ-potential are factors correlated with bacterial adhesion to the epithelial cells, which are guided by charge and hydrophobicity of the bacterial surface.

The presence of surface layers being reportedly linked to tolerance toward stresses (do Carmo et al., 2018), we decided to investigate the impact of such a mutation on the CB129Δ*slpB* strain tolerance toward stress challenges that are relevant for the selection of new probiotics. The ability to survive acid stress in the stomach and bile salts stress in the duodenum during the passage through the digestive tract, is important for probiotic interaction with the host (Rabah et al., 2017). Accordingly, *in vitro* assays can be used to simulate digestive stresses, mimicking the exposure to acidic conditions (pH 2.0) or to biliary salts (1 g.L^−1^) (Jan et al., 2000). For *P. freudenreichii*, commonly used as a cheese starter, the heat stress tolerance constitutes a relevant technological ability of this strain (Rosa do Carmo et al., 2017). Overall, we observed a large decrease in tolerance to the environmental stresses, confirming a role of SlpB in toughness. In the guanidine-extracted proteins of the mutant strain, the chaperones and heat shock proteins, DnaK1, DnaK2, ClpB 2, GroE1, and GroE2 were found. Inside the cell, they are responsible for protein folding and are correlated to acid and bile adaptation (Leverrier et al., 2005; Gagnaire et al., 2015). Here, they were found at the surface of the CB129Δ*slpB* mutant, which was more susceptible to extreme acid stress and temperature, compared to wild type strain. Previous work showed that *L. acidophilus* ATCC 4356 adapts to harsh environments by increasing the expression of the s-layer SlpA protein upon bile, acidic pH and heat stress exposition (Khaleghi et al., 2010; Khaleghi and Kasra, 2012). Moreover, changes in the cell surface properties could alter the transmembrane protein complex responsible for the extrusion of protons from the cytoplasm, which are responsible for surviving environmental stresses (Ruiz et al., 2013; Rosa do Carmo et al., 2017).

Profound modifications of *P. freudenreichii* physiology and surface properties suggested that modifications, wider than the disappearance of a single protein, occurred as a result of *slpB* gene inactivation. To understand this impact of the mutation, a comparative proteomic analysis was performed to identify significant alterations in the whole proteome profile of the mutant strain, using label-free quantitative proteomic analysis. Prediction of sub cellular localization using the SurfG+ tool (Barinov et al., 2009) evidenced changes in all the categories (CYT, MEM, PSE and SEC) in the differential proteome of CB129Δ*slpB*. In addition, differential proteome was functionally classified using COG, showing a functional implication of differential proteins in cellular processes such as signaling, information storage, processing, and metabolism. Specifically, this study showed that the moonlighting enolase and NlpC/P60 are both exported (Frohnmeyer et al., 2018), as it was recently observed in the cutaneous *Propionibacterium acnes* strain (Jeon et al., 2017). These moonlighting proteins were downregulated in CB129Δ*slpB*. Interestingly, in the *Bifidobacterium* and *Lactobacillus* genera, moonlighting proteins, such as enolase, also play a role in immunomodulation and adhesion (Sánchez et al., 2010; Kainulainen and Korhonen, 2014; Vastano et al., 2016). Furthermore, in the PPI network we observed high interactions between the downregulated Enolase (PFCIRM129_06035), reportedly involved in human gut colonization and stress adaptation (Ruiz et al., 2009), with other proteins involved in several other processes, including metabolism and DNA repair. Moreover, all surface layer-associated proteins SlpA, SlpD, SlpE, and InlA were downregulated in CB129Δ*slpB*. These proteins form a protective layer on the surface of the bacteria, and have been associated with environmental stress tolerance (Fagan and Fairweather, 2014). As seen previously, a decreased amount of these proteins could be directly associated with stress susceptibility and with altered hydrophobicity. SLAPs can directly influence these properties (Pum et al., 2013), and consequently alter adhesion to epithelial cells (do Carmo et al., 2017).

We performed the complete genome DNA sequencing of the CB129Δ*slpB*, which, in turn, allowed us to evaluate whether the *slpB* gene disruption had major consequences on the mutant strain genome. The *slpB* gene is not part of an operon, which suggests that homologous recombination using the suicide plasmid pUC:Δ*slpB*:*Cm*R (do Carmo et al., 2017) did not affect the expression of upstream and downstream genes. Analysis of the genetic context, upstream and downstream, revealed that the homologous recombination process was site-specific, and not affecting other genes in the genome of the mutant strain CB129Δ*slpB*. However, we were unable to evaluate possible rearrangements in the genome of CB129Δ*slpB*, which could have affected the transcription of other genes. Therefore, more studies are necessary to explore whether any probiotic potential was lost after the single mutation of the *slpB* gene in *Propionibacterium freudenreichii* CIRM-BIA 129 strain.

## Conclusion

This study evidenced the pleiotropic impact of the surface layer protein *slpB* mutation in the probiotic strain *Propionibacterium freudenreichii* CIRM-BIA 129 in relation to its physicochemical proprieties, stress challenges, surfaceome and whole cell quantitative proteome. It confirmed the key role of SLPs and strongly suggests that expression of specific ones, such as *P. freudenreichii* SlpB, should be used as criteria for selecting strains with probiotic potential.

## Author contributions

FC performed *in vitro* assays, microscopy, proteomic assays and data interpretation. WS, FP, GT, and ROC performed proteomic assays, data interpretation and bioinformatics analyses. BC, EO, and SS performed *in vitro* assays. II and HR data interpretation. EF performed PPI network. CC performed microscopy. MC, AC, and RS performed genomics and data interpretation. VA, GJ, HF, and YL contributed to the supervision, analysis, and interpretation of data and were major contributors to revising the manuscript. All authors contributed in writing the manuscript.

### Conflict of interest statement

The authors declare that the research was conducted in the absence of any commercial or financial relationships that could be construed as a potential conflict of interest. The reviewer MAS declared a shared affiliation, with no collaboration, with one of the authors, WS, to the handling editor at time of review.

## Supplementary material

Figure S1 Verification of assembly error by read mapping. The plasmid pUC:Δ*slpB*:*Cm*R was not inserted in the *slpB* gene during de novo genome assembly. The read mapping on the *slpB* gene shows misalignments upstream and downstream insertion site, confirming the assembly error. The read mapping was performed using in CLC Genomics Workbench 7.0.

Figure S2 Assembly curation and validation by read mapping. The manual insertion of plasmid pUC:Δ*slpB*:*Cm*R in the *slpB* gene was validated by read mapping. The correct read alignments upstream **(A)** and downstream **(B)** the plasmid validate the manual insertion. The read mapping was performed using in CLC Genomics Workbench 7.0.

Table S1 Total list of proteins identified in the core-proteome of CB 129 wild-type and CB129Δ*slpB*.
