# Supplementary figures and images for: Mutation of the Surface Layer Protein SlpB Has Pleiotropic Effects in the Probiotic Propionibacterium freudenreichii CIRM-BIA 129
Source: Front Microbiol. 2018 Aug 17;9:1807. doi: 10.3389/fmicb.2018.01807 (PMC6107788; doi:10.3389/fmicb.2018.01807)

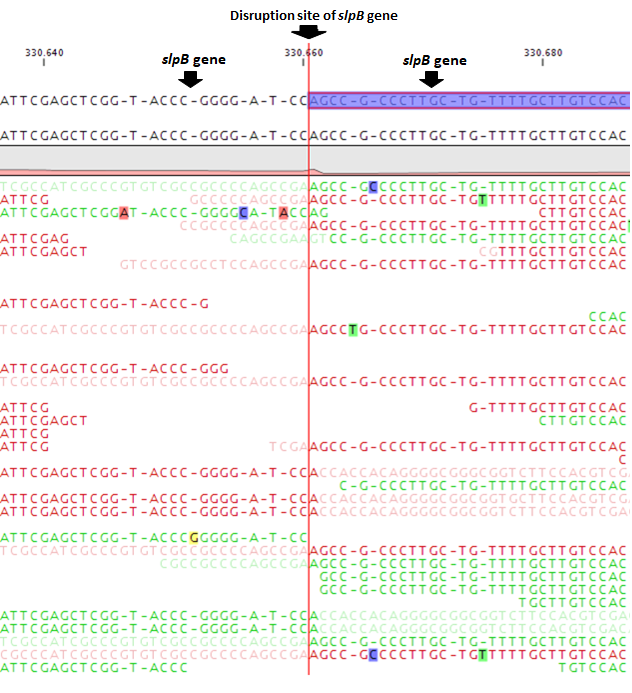

Supplement: Figure S1 — Verification of assembly error by read mapping. The plasmid pUC:ΔslpB:CmR was not inserted in the slpB gene during de novo genome assembly. The read mapping on the slpB gene shows misalignments upstream and downstream insertion site, confirming the assembly error. The read mapping was performed using in CLC Genomics Workbench 7.0. [file Image_1.TIF]

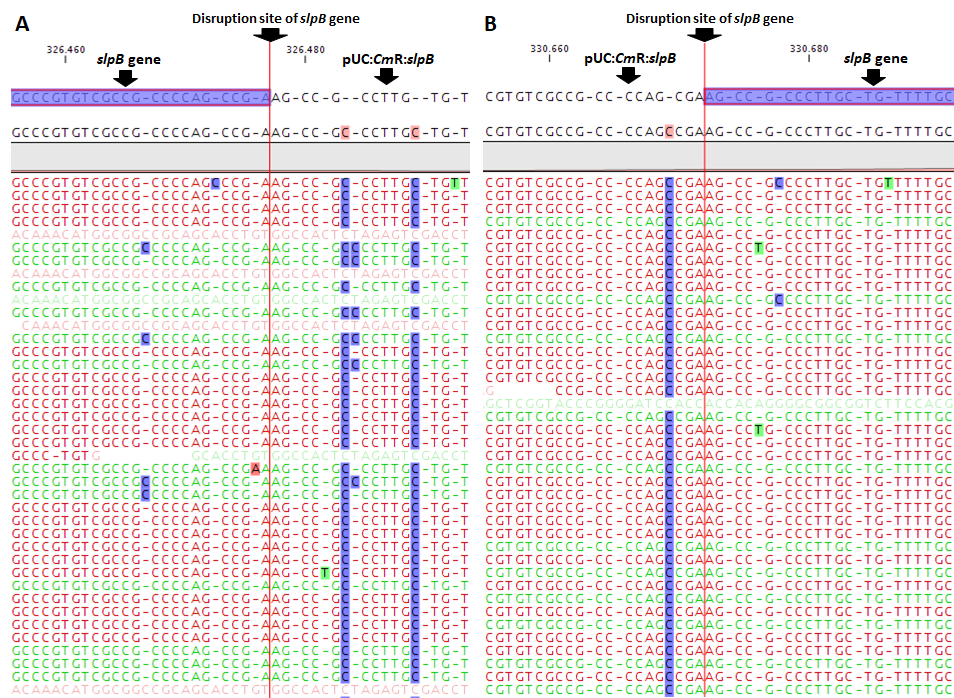

Supplement: Figure S2 — Assembly curation and validation by read mapping. The manual insertion of plasmid pUC:ΔslpB:CmR in the slpB gene was validated by read mapping. The correct read alignments upstream (A) and downstream (B) the plasmid validate the manual insertion. The read mapping was performed using in CLC Genomics Workbench 7.0. [file Image_2.TIF]
